# Plastome evolution and phylogenomic insights into the evolution of *Lysimachia* (Primulaceae: Myrsinoideae)

**DOI:** 10.1186/s12870-023-04363-z

**Published:** 2023-07-14

**Authors:** Tong-Jian Liu, Shu-Yan Zhang, Lei Wei, Wei Lin, Hai-Fei Yan, Gang Hao, Xue-Jun Ge

**Affiliations:** 1 Key Laboratory of Plant Resources Conservation and Sustainable Utilization, South China Botanical Garden, Chinese Academy of Sciences, Guangzhou, China; 2 South China National Botanical Garden, Guangzhou, 510650 China; 3 College of Life Sciences, South China Agricultural University, Guangzhou, China; 4 University of Chinese Academy of Sciences, Beijing, China

**Keywords:** Primulaceae, *Lysimachia*, Plastid genome, Phylogeny, Conflict signature

## Abstract

**Background:**

*Lysimachia* L., the second largest genus within the subfamily Myrsinoideae of Primulaceae, comprises approximately 250 species worldwide. China is the species diversity center of *Lysimachia*, containing approximately 150 species. Despite advances in the backbone phylogeny of *Lysimachia*, species-level relationships remain poorly understood due to limited genomic information. This study analyzed 50 complete plastomes for 46 *Lysimachia* species. We aimed to identify the plastome structure features and hypervariable loci of *Lysimachia*. Additionally, the phylogenetic relationships and phylogenetic conflict signals in *Lysimachia* were examined.

**Results:**

These fifty plastomes within *Lysimachia* had the typical quadripartite structure, with lengths varying from 152,691 to 155,784 bp. Plastome size was positively correlated with IR and intron length. Thirteen highly variable regions in *Lysimachia* plastomes were identified. Additionally, *ndhB, petB* and *ycf2* were found to be under positive selection. Plastid ML trees and species tree strongly supported that *L. maritima* as sister to subg. *Palladia* + subg. *Lysimachia* (Christinae clade), while the nrDNA ML tree clearly placed *L. maritima* and subg. *Palladia* as a sister group.

**Conclusions:**

The structures of these plastomes of *Lysimachia* were generally conserved, but potential plastid markers and signatures of positive selection were detected. These genomic data provided new insights into the interspecific relationships of *Lysimachia*, including the cytonuclear discordance of the position of *L. maritima*, which may be the result of ghost introgression in the past. Our findings have established a basis for further exploration of the taxonomy, phylogeny and evolutionary history within *Lysimachia*.

**Supplementary Information:**

The online version contains supplementary material available at 10.1186/s12870-023-04363-z.

## Background

Lysimachieae is the second largest tribe within the subfamily Myrsinoideae of Primulaceae, mainly distributed in temperate and subtropical climates of the Northern Hemisphere, although it is found worldwide [1]. The tribe is traditionally considered a monophyletic group with six genera, namely *Lysimachia* L*.*, *Anagallis* L., *Trientalis* L., *Glaux* L., *Asterolinon* Hoffmanns. & Link, and *Pelletiera* A. St.-Hil. These genera were characterized by the pattern of capsule dehiscence, number of corolla lobes, and corolla color [2, 3]. While the morphological distinctiveness of the genera within the tribe is relatively easy to recognize, the morphological delimitation among the genera is not clear in nature. *Lysimachia* s.s. is the largest and most morphologically diverse genus in the tribe, with approximately 180 species, and is segregated into six subgenera: subg. *Idiophyton*, subg. *Heterostylandra*, subg. *Lysimachia*, subg. *Lysimachiopsis*, subg. *Naumburgia* and subg. *Palladia* [4–6]. However, the monophyly of *Lysimachia* s.s. has been questioned based on morphology and molecular data [2, 7–10]. For example, *Anagallis arvensis* L. is strikingly similar to *L. nemorum* L., *L. serpyllifolia* Schreb. and a few other *Lysimachia* species, being distinguished solely by color of the corolla and mode of capsule dehiscence [7, 9, 11]. Molecular phylogenetic results confirmed the above finding and even found that these satellite genera (*Anagallis*, *Trientalis*, *Glaux*, *Asterolinon*, and *Pelletiera*) were all embedded within *Lysimachia* s.s. [2, 7, 8, 10, 12–14]. Consequently, the most recent treatment of the tribe synonymized all of these genera with *Lysimachia*, and formed a newly circumscribed *Lysimachia* s.l. with approximately 250 species [11, 15]. Previous molecular phylogenetic studies further detected at least 11 main clades within *Lysimachia* s.l. [2, 8, 10, 16], which were broadly consistent with the former subgenera of *Lysimachia* s.s. and previously described genera, and recognized four new clades from subg. *Lysimachia* of *Lysimachia* s.s. (i.e., Christinae clade, Vulgaris clade, Nemorum clade and Andina clade) [10]. The newly circumscribed genus *Lysimachia* were used in this study.

China is widely recognized as the species diversity center of *Lysimachia*, with eight clades/subgenera (i.e., subg. *Idiophyton*, Christinae clade of subg. *Lysimachia*, Vulgaris clade of subg. *Lysimachia*, subg. *Palladia*, subg. *Naumburgia*, *Glaux* clade*, Trientalis* clade, and *Anagallis* clade) [5, 10]. The first three subgenera all exceed 35 species and have undergone rapid speciation since the Middle Miocene [10]. However, the species-level relationships within Chinese *Lysimachia* remain controversial. For instance, *Lysimachia maritima* (L.) Galasso, Banfi & Soldano (= *Glaux maritima* L.) was recovered as sister to subg. *Palladia* + subg. *Lysimachia* (Christinae clade) with weak support based on 10 plastid markers and one ITS [10]. If only based on ITS data, *L. maritima* was recovered as sister to subg. *Palladia* [12] or subg. *Lysimachia* (Christinae clade) [8]. These uncertainties in phylogenetic relationships within *Lysimachia* are likely due to limited genetic markers and/or taxon sampling employed in previous studies, rapid evolution history, incomplete lineage sorting (ILS), and hybridization. Additionally, the magnitude of infraspecific variation in many widespread species (e.g. *L. fortunei* Maxim.) or the status of taxonomic entities in some taxa (e.g. *L. coreana* Nakai) lack detailed studies.

The plastid is an essential organelle in plant cells, playing a crucial role in plant growth and development [17]. The majority of complete plastomes in plants have a typical tetrad structure, consisting of a large single copy (LSC), a small single copy (SSC), and two copies of inverted repeats (IR_A_ and IR_B_) [18]. Due to its conserved genome structure, moderate size, and stable gene content, the plastome is a powerful marker for elucidating complex evolutionary relationships at various taxonomic levels of plants [19–21]. The advancement of high-throughput sequencing technology and the reduced cost of sequencing have enabled the widespread use of plastome phylogeny in plants. However, only seven plastomes of *Lysimachia* have been reported and/or released on GenBank (Table S1) [22–26], leading to a lack of progress in the phylogenomics of *Lysimachia*.

In this study, we assembled the complete plastomes for 43 *Lysimachia* species and combined them with seven available *Lysimachia* plastomes in GenBank. The objectives of this study were to (1) identify the plastome structure variation and features of *Lysimachia*; (2) determine the hypervariable loci for the identification of *Lysimachia*; (3) reconstruct the phylogenetic tree to elucidate *Lysimachia*’s relationships; and (4) examine the phylogenetic conflict signals for some *Lysimachia* species. Ultimately, this study should contribute to a better understanding of the rapid evolution of *Lysimachia* based on plastome data.

## Results

### Basic characteristics of plastomes in *Lysimachia*

**Fig. 1:**
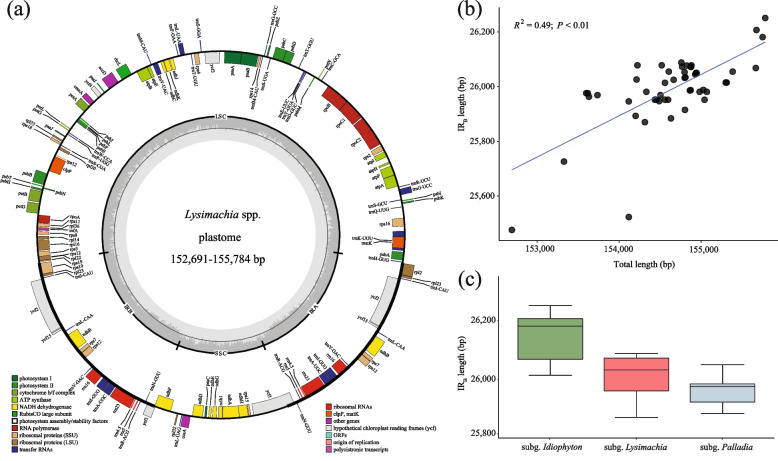
The plastomes and their expansion/contraction of IR among *Lysimachia* species. **a** plastome map of *Lysimachia*. The genes belonging to different functional groups are shown in different colors, which are shown on the bottom left. Genes inside and outside of the external circle are transcribed in clockwise and counterclockwise directions, respectively. The inner circle represents the quadripartite structure, with two copies of the inverted repeat (IR_A_ and IR_B_), an LSC, and an SSC region in black with GC content in dark gray and AT content in light gray. **b** Correlation of IR length and *Lysimachia* plastome size. **c** Boxplot of the IR length in three subgenera of *Lysimachia*

In this study, fifty whole plastomes of *Lysimachia* were analyzed (Table S2). The length of these plastomes ranged from 152,691 bp (*L*. *mauritiana* Lam.) to 155,784 bp (*L*. *capillipes* Hemsl.). All these plastomes contained the typical quadripartite structure, consisting of a pair of IRs (25,476 bp–26,251 bp) separated by the LSC region (83,676 bp–85,520 bp) and SSC region (17,844 bp–18,203 bp) (Fig. 1a; Table S2). The characteristics of these plastomes are conserved in terms of gene content and GC content (Table S2). The total GC content among these plastomes varied from 36.9% to 37.1%. Each plastome encoded 133 predicted functional genes, of which 19 were duplicated in the IR regions (Table S2). Among the unique genes, there were 80 protein-coding genes, four rRNA genes, and 30 tRNA genes. Within the IR, eight protein-coding genes, seven tRNA genes, and all four rRNA genes were completely duplicated. Additionally, 11 genes possessed a single intron (i.e., *atpF*, *petB*, *petD*, *ndhA*, *ndhB*, *rpoC1*, *rpl2*, *rps12*, *rps16*, *trnA-UGC* and *trnI-GAU*), and two genes (*ycf3* and *clpP*) contained two introns (Table S3).

Across the 50 *Lysimachia* species, the *ycf1* gene overlapped in the SSC-IR_A_ junction region, ranging from 971 bp (*L. monelli* (L.) U.Manns & Anderb.) to 1,014 bp (*L. klattiana* Hance) in the IR_A_ region with the exception of *L. stenosepala* Hemsl., whose *ycf1* gene (4,428 bp) was located entirely in the SSC region (Fig. S1). Additionally, a duplicated pseudogene *ycf1* was present in the IR_B_ region. The *trnH* gene, with a length of 68–75 bp, was conservatively distributed on the right side of the LSC-IR_A_ region. A significant correlation was observed between the size of the plastome and the IR length among *Lysimachia* species (*R*^2^ = 0.49, *P* = 0.01; Fig. 1b). Besides, a strong relationship between plastome sizes and intron length, while relationship between plastome sizes and IGS length was not significant (Fig. S2). The species were then divided into three subgenera for IR region analysis (Fig. 1c). Subg*. Palladia* was found to have a relatively small IR size (Fig. 1c), with the *rps19* gene of 150 bp entirely embedded into the LSC region, with a gap ranging from 51 bp (*L. pentapetala* Bunge) to 149 bp (*L. auriculata* Hemsl.) (Fig. S1). In contrast, the LSC-IR_B_ regions of subg. *Idiophyton*, subg*. Lysimachia* (i.e., clade I, referred to as the “Christinae clade” in text; see the phylogenetic results below) and subg. *Lysimachia* (i.e., clade IV, referred to as the “Vulgaris clade” in the text; see the phylogenetic results below) were located within the *rps19* gene (237 bp), resulting in the presence of *rps19* duplication in the IR_A_ region. The IR_B_/SSC boundary was relatively conserved in all *Lysimachia* taxa, with the *ndhF* gene crossing over to the IR_B_ region (Fig. S1).

### Comparative genome analysis and mutation hotspot identification

No gene rearrangements were identified in *Lysimachia* based on Mauve alignment (Fig. S3), indicating that their genome structure was highly conserved. The mVISTA-based identity plot also exhibited a high degree of conservation in genome structure, gene order, and gene content across all *Lysimachia* plastomes (Fig. S4). Variability was higher in the LSC and SSC regions than in the two IR regions. Furthermore, more variable characteristics were observed in the noncoding regions than in the coding regions. Specifically, distinct sequence variations in intergenic regions included *rps16-trnQ*-*UUG, trnG*-*UCC-trnR*-*UCU, trnY*-*GUA-trnT*-*GGU, ndhC-trnV*-*UAC, petA-psbJ, rps12-rpl32* and *ccsA-ndhD*. The rRNA genes were highly conserved. Several protein-coding genes were highly conserved (e.g., *rpoB, psbD, psaA, psaB, psbC, psbB* and *rpl23*), while others (e.g., *trnR, trnH* and *ycf1*) had slightly more variation.

**Fig. 2:**
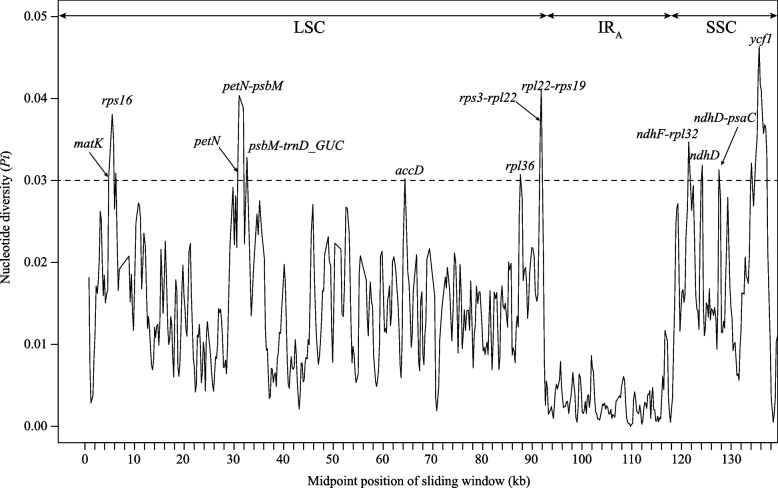
Comparison of nucleotide diversity (*Pi*) values in *Lysimachia* plastomes. The X-axis shows the position of the midpoint of sliding windows (kb), while the Y-axis indicates the nucleotide diversity of each window

Sliding window analysis across all *Lysimachia* plastomes revealed that the *Pi* values in the IR regions were more conserved than those in the single copy (SC) regions (Fig. 2). The *Pi* values ranged from 0 to 0.0464, with an average of 0.0113. The average *Pi* values of the LSC, SSC, and IR regions were 0.0144, 0.0210, and 0.0043, respectively. Highly variable hotspots (*Pi* > 0.03) were identified in *matK*, *rps16*, *petN*, *petN-psbM*, *psbM-trnD*-*GUC*, *accD*, *rpl36*, *rps3*-*rpl22*, *rpl22-rps19*, *ndhF-rpl32*, *ndhD*, *ndhD*-*psaC* and *ycf1*. Nucleotide diversity among the four clades (with more than two species) was also analyzed. In subg. *Palladia* (clade II), their *Pi* values varied from 0 to 0.0320, with an average value of 0.0063 (Fig. S5a). Intergenic regions (i.e., *trnK*^*UUU*^-*rps16*, *petN*-*psbM* and *trnT*^*UGU*^-*trnF*^*GAA*^) and coding regions (i.e., *petA*, *rpl32* and *ycf1*) had relatively high sequence divergence. The average nucleotide diversity (*Pi*) of subg. *Idiophyton* (clade VI) was 0.0049 with a range of 0 to 0.0280, whereby their LSC, SSC, and IR regions had average *Pi* values of 0.0084, 0.0064, and 0.0014, respectively (Fig. S5b). For the Christinae clade of subg*. Lysimachia* (clade I), *rps3-rpl22* exhibited the greatest variation, with a maximum value of 0.2757 (Fig. S5c)*.* Other relatively highly variable regions (i.e., *trnK*-*UUU-rps16*, *rps16-trnQ*-*UUG*, *petN-psbM*, *accD*, *psab-petL*, *ndhF-rpl32*, *ndhD* and *ycf1*) were identified. The *Pi* values of the Vulgaris clade of subg. *Lysimachia* (clade IV) had a range of 0–0.0427, with an average value of 0.0089 (Fig. S5d). There were six highly variable regions (*Pi* > 0.03), namely, *trnH*-*GUG*, *trnS-GCU-trnR-UCU*, *rpl16*, *ndhD*, *psaC* and *ycf1*.

### Selective pressure analysis

**Fig. 3:**
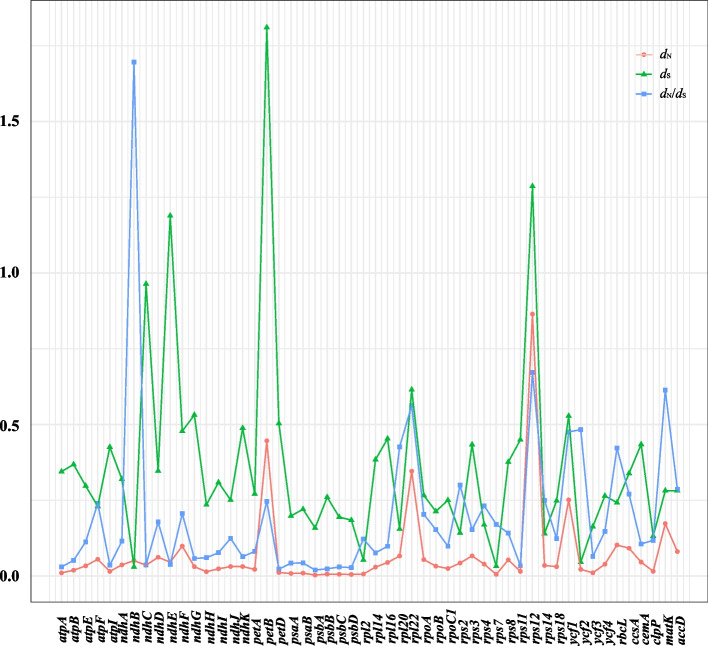
Comparisons of *d*_N_, *d*_S_, and *d*_N_/*d*_S_ of the protein-coding genes in *Lysimachia*

The ratio of nonsynonymous (*d*_N_) to synonymous (*d*_S_) mutations is a powerful tool in selection pressure analysis. A *d*_N_/*d*_S_ (ω) ratio less than 1 signifies negative selection, while a ω ratio greater than 1 indicates positive selection. Protein-coding genes shorter than 300 bp were filtered out from this analysis*.* In *Lysimachia* (excluding *L. monelli* and *L. europaea* (L.) U. Manns & Anderb.), 52 protein-coding genes were identified, and the results showed that *d*_S_ values were greater than *d*_N_ values in most genes, indicating purifying selection (Fig. 3). However, the *ndhB* gene was under positive selection with a ratio of 1.6956 (ω > 1) (Fig. 3).

To further understand the adaptive evolution of *Lysimachia*, this analysis was applied to three main clades. The subg. *Lysimachia* (clade IV, Vulgaris clade) was excluded due to its few species (*L*. *vulgaris* L*.* and* L*. *davurica* Ledeb*.*). A total of 53 genes were used in subg. *Palladia*, displaying *d*_N_ values of 0–0.6511 and *d*_S_ values of 0.0104–2.1268 (Fig. S6a). The *petB* gene was the only one to present positive selection with a ω ratio of 1.0043. In subg. *Idiophyton*, the ω ratio of 49 protein-coding genes was less than 1, indicating purifying selection on these genes (Fig. S6b). The *ycf2* gene had the highest ω ratio of 0.9241 in subg. *Idiophyton*. Fifty-one shared protein-coding genes were analyzed in subg*. Lysimachia* (clade I, Christinae clade), and the results demonstrated that only the *ycf2* gene experienced positive selection with a ω ratio of 1.3690, while the other genes were under purifying selection (Fig. S6c).

### Phylogenetic analysis based on plastomes and nuclear rDNA

**Fig. 4:**
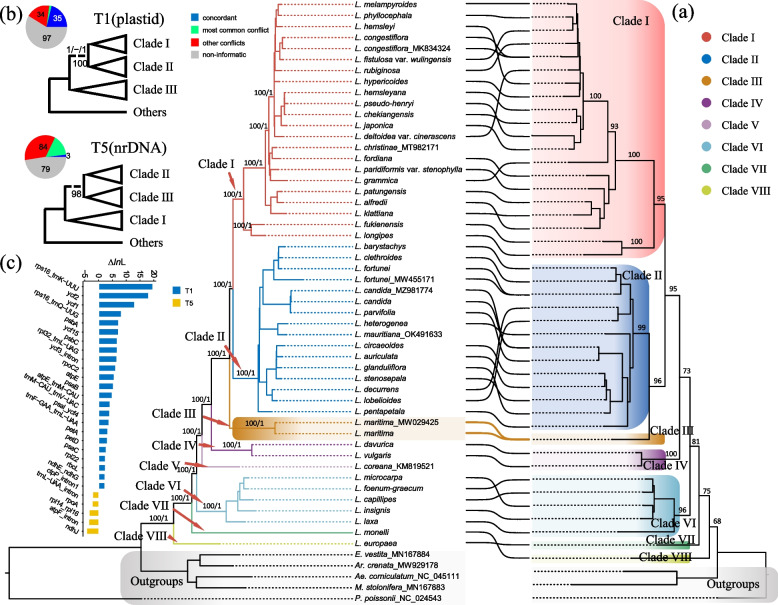
Conflict signals between plastid and nrDNA phylogenies in *Lysimachia*. **a** The tanglegram of *Lysimachia* shows cytonuclear conflicts between the whole plastid ML tree (T1; left) and nrDNA ML tree (right). Eight major clades within *Lysimachia* are indicated by different colors, and the tangles among the group of *L. maritima* are highlighted. **b** Two hypotheses between two constraint phylogenies according to conflicting cytonuclear topologies using whole plastid datasets and the ratio of gene tree supports, conflicts and non-information. The focal branches we tested in two constraint topologies are shown with dotted lines. The bootstrap supports are shown above the focal branch, and three quartet metrics from quartet sampling analysis are shown below the focal branch. The colors in the legend indicate concordant, most common conflict, other conflicts, and non-informatics, respectively. **c** The difference in log-likelihoods (Δ*ln*L) of two hypotheses (T1 and T5) for each locus calculated in RAxML. The positive values of Δ*ln*L indicate T1-supported phylogenetic signals, while negative values support the T2 hypothesis. Only the genes with significant phylogenetic signals (Δ*ln*L > 2) are shown on the bar plot. Δ*ln*L values of T1 and T5 were showed with dark blue and yellow, respectively

In this study, the plastome sequences of 50 *Lysimachia* species were used for phylogenetic relationship reconstruction. Maximum likelihood topologies inferred from the concatenated datasets of whole plastome loci, protein-coding genes (PCGs), intergenic spacers (IGS), and introns (T1-T4; Fig. 4a; Figs. S7-9) were largely similar, with only a few short internal branches having low bootstrap supports (e.g., the positions of *L. rubiginosa* Hemsl*.*,* L*. *deltoidea* var. *cinerascens* Franch.) (Figs. S7-9). The MQSST species tree inferred by ASTRAL had a convergent topology compared to the ML trees (Fig. 4a; Fig. S10).

*Lysimachia* taxa were recovered as monophyletic with full support, and eight major clades in all four plastid ML trees and the ASTRAL species tree were fully supported (BS = 100; LPP = 1) (Fig. 4a; Fig. S10). Clade VIII contained *L. europaea* (belonging to the former genus *Trientalis*), which was resolved as sister to the remaining *Lysimachia* taxa, with clade VII (*L. monelli*, belonging to the former genus *Anagallis*) sister to clades I-VI. All datasets strongly supported subg. *Idiophyton* was formed as monophyletic group (clade VI), positioned as sister to clades I-V. Clades V (*L*. *coreana*) and IV (containing *L*. *davurica* and *L*. *vulgaris* both belong to subg. *Lysimachia*; Vulgaris clade) was placed as a successive sister to clades I-III. Clade I contained the remaining taxa of subg. *Lysimahcia* (Christinae clade). Clade II contained the all species from subg. *Palladia*. Two individuals of *L*. *maritima* clustered into clade III (belonging to the former monotypic genus *Glaux*). These plastid datasets strongly supported a sister relationship between clade I and clade II. Our results demonstrated that subg. *Palladia* (clade II) and subg. *Idiophyton* (clade VI) was monophyletic, while subg. *Lysimachia* (including clade I, clade IV and clade V) was paraphyletic (Fig. 4a; Fig. S10).

The nuclear rDNA (nrDNA) topology was largely congruent with that of trees based on plastomes (Fig. 4a). Upon comparison of phylogenetic trees, we identified several conflicting signals, which were mainly concentrated within clade I and II. These conflicting signals were unstable, as they occur at positions with short branch lengths and low supports (Fig. 4a; Fig. S7-10). However, we observed a significant difference in the position of *L. maritima* between the two phylogenetic trees. The nrDNA phylogeny strongly supported (BS = 96) a sister relationship of clade III (*L*. *maritima*) and clade II, generating an alternative hypothesis (Fig. 4a). To further investigate this positional conflict of clade III in *Lysimachia*, we tested the topology of five ML trees (T1—T5) using IQ-TREE v2.2.0. T5 was the constrained plastid tree, conforming to the alternative hypothesis of Clade III in nrDNA phylogeny (Fig. 4a). The AU, wKH and wSH tests revealed that the whole plastid tree (T1), the PCG tree (T2) and the IGS tree (T3) all passed these tests, whereas the intron (T4) and constrained tree (T5) were statistically rejected (Table S4).

### Incongruence assessment and phylogenetic signal heterogeneity

We identified incongruences in the phylogeny of the optimal plastid tree (T1) using quartet sampling and phyparts at both internal branch and terminal (rogue taxa) levels. Our quartet sampling revealed an average the quartet informativeness (QI) value of 0.95 for all branches, with 84.6% of them strongly supported (the quartet concordance (QC) > 0.2; Fig. S11). Low quartet concordance (QC < 0.2) was observed for short branches with low support values (BS and LPPs; Figs. S7-11). However, one group, containing *L. fortunei*, *L. barystachys* Bunge and *L. clethroides* Duby, showed lower quartet concordance (QC < 0.2) at long branches with high support (BS = 100; LPP = 1) (Figs. S7-11). Our quartet sampling analysis fully or strongly supports the relationships of Clade III (*L. maritima*) as sister to Clades I and II (Fig. S11). The phyparts analysis also showed that all eight clades (I—VIII) had the most concordant support at the gene tree level (Fig. S12), although two dominant alternative topologies (containing ca. 50% conflict gene trees; Fig. S12) were found at the ancestor node of clades I and II. Notably, 35 gene trees supported the relationship of Clade III (*L. maritima*) sister to Clades I and II, although 34 gene trees disagreed with this pattern. Therefore, both quartet sampling and phyparts analyses support Clade III (*L. maritima*) as a sister to Clades I and II.

The optimal plastid tree (T1) was selected and combined with the constrained tree (T5) to further test the relationship among clades I-III. We extracted 156 loci and evaluated the support of each locus for two topologies (T1 and T5). T1 was strongly supported by 23 loci (e.g., *rps16_trnK-UUU, ycf2, ycf1, rps16_trnQ-UUG, psbA, ycf15, psbC, rpl32_trnL-UAG, ycf3_intron, rpoC2, atpE, psaB, atpE_trnM-CAU, trnM-CAU_trnV-UAC, psaI_ycf4, trnF-GAA_trnL-UAA, petA, petD, psaC, rpl22, rbcL, ndhE_ndhG* and *clpP_intron1*; their absolute △*ln*L > 2). T5 was supported by only five loci (e.g., *trnL-UAA_intron, rpoA, rpl14_rpl16, atpF_intron* and *ndhJ*; their absolute △*ln*L > 2) (Figs. 4b, c; Table S5). To assess whether a small number of genes dominate the phylogenetic estimation, we removed the top five loci with the largest absolute △*ln*L from the concatenated whole plastome supermatrix. The phylogenetic tree inferred without the top five plastid loci was concordant with the original plastid tree, indicating the robustness of the phylogenetic relationship among the three clades (clades I-III).

## Discussion

### Plastome structure variation and selective evolution in *Lysimachia*

The content and structure of genomes are closely linked to speciation and the ability of species to adapt to changing environments. In plants, the plastome plays a crucial role in growth and development, as it encodes key proteins involved in photosynthesis and other metabolic processes. However, the relationship between the evolutionary patterns of the plastome of *Lysimachia* and its rich species diversity and diverse habitats remains largely unknown due to the lack of sufficient plastomic data. To address this gap, we conducted a study in which we sequenced and assembled a large number of high-quality complete plastomes for this plant group, providing an unprecedented opportunity to conduct in-depth comparative genomics studies and attempt to unravel its evolutionary history.

Previous studies have shown that plastomes in *Lysimachia* possess a typical tetrad structure and are highly conserved in terms of genome size, structure, GC content, and gene composition [23–26]. Our study, based on 50 complete plastomes of *Lysimachia*, partially confirmed this finding. While GC content and gene composition were relatively conserved, plastome size in *Lysimachia* varied from 152,691 bp (*L. mauritiana*) to 155,784 bp (*L. capillipes*). Previous studies have identified three factors that contribute to the variation in plastome size, including expansion/contraction of the IR regions, gene loss and additional gene duplications outside of the IR, and the sizes of introns and intergenic spacer regions [27]. Our findings suggest that expansion/contraction of the IR regions is a major contributor to plastome sequence variation in *Lysimachia*, as supported by the positive relationship between genome size and IR length among species in this study (Fig. 1b). In contrast, gene loss and additional gene duplications outside of the IR do not appear to be major factors in *Lysimachia*, as their gene content and numbers are highly conserved (Table S2). Finally, we found that introns contribute to the variation in plastome size of *Lysimachia*, but intergenic spacer regions (IGS) do not (Fig. S2).

Our study revealed that plastome size has phylogenetic significance, with subg. *Idiophyton* having the largest plastome size, followed by subg. *Lysimachia*, while subg. *Palladia* has the most compact plastome (Fig. 1c; Table S2). Interestingly, *L. mauritiana*, a member of subg. *Palladia*, has the smallest plastome size (152,691 bp). This species is a sea perennial plant living along beaches and maritime rock crevices of East and Southeast Asia, Pacific Islands, and Indian Ocean Islands [1]. Generally, heterotrophic organisms tend to have smaller genome sizes due to the loss of functional genes in the plastome [27]. However, our findings suggest that plastome size is associated with IR expansion/contraction and intron size, rather than overall genome complexity. The relationship between environmental stress factors and IR expansion/contraction and intron size requires further investigation.

To examine the adaptation of organisms to diverse environments, we analyzed whether coding genes are subject to selection using *d*_N_*/d*_S_ (ω) ratios. Our results showed that *ndhB* (ω = 1.6956) in *Lysimachia*, *petB* (ω = 1.0043) in subg. *Palladia*, and *ycf2* (ω = 1.3690) in subg. *Lysimachia* (Christinae clade) were under positive selection, while all protein-coding genes in the plastomes of subg. *Idiophyton* were under negative selection with ω < 1. The *ndhB* gene encodes a subunit of NADPH dehydrogenase, which plays a crucial role in photosynthetic electron transport and dark respiration of photosynthesis [28]. The *petB* gene encodes Cytochrome b6 of the Cyt b6/f complex, a subunit of the photosynthetic apparatus [29], while the *ycf2* gene encodes a 2-MD heteromeric AAA-ATPase complex, which is essential for plant viability and ATP production in chloroplasts in darkness [30]. All these positively selected genes are relevant to ecological adaptation to light preference. Most *Lysimachia* species are shade-tolerant herbs, but some taxa, such as *L. mauritiana*, *L. fortunei*, and *L. maritima*, prefer to grow in open areas and are adapted to sunny habitats [1]. These habitat shifts may have facilitated the divergence of the functional genes mentioned above. Although our study had limited samples, the detected signatures of adaptations to light environment shifts provide valuable clues for understanding the evolution of *Lysimachia* plastomes in response to diverse ecological habitats.

### Potential DNA markers for *Lysimachia* identification

Plastid markers have been a powerful tool for species identification and phylogenetic inferences in many phylogenetic studies. However, only a few plastid markers have been used in barcoding and phylogenetic inference in *Lysimachia* [2, 8, 10, 12, 31, 32]. In this study, 20 variable regions in *Lysimachia* were identified as potential DNA markers, including *rps16*, *rps16*-*trnQ-UUG*, *trnG*-*UCC-trnR*-*UCU, trnY*-*GUA-trnT*-*GGU, ndhC-trnV*-*UAC, petA-psbJ, rps12-rpl32, ccsA-ndhD*, *petN*-*psbM*, *psbM*-*trnD-GUC*, *accD*, *rpl36*, *rpl22*-*rps19*, *ndhD*, *ndhF*-*rpl32*, *ycf1*, *petN*, *matK*, *rps3*-*rpl22* and *ndhG*-*ndhI*. Of these, only two molecular markers (*rps16* and *matK*) have been used in previous studies [2, 8, 10, 31, 32]. Additionally, several highly variable regions (i.e., *rps16*-*trnQ-UUG*, *petN*-*psbM*, *accD*, *rpl22*-*rps19*, *ndhF*-*rpl32*, *ccsA*-*ndhD* and *ycf1*) were also found in the Myrsinaceae s.str clade in Primulaceae [26], suggesting that these markers can be used as important genetic resources for future studies on the evolution and diversity of Primulaceae. Notably, several highly variable regions found in this study (i.e., *rps16*, *rps16*-*trnQ-UUG*, *ccsA*-*ndhD*, *petA*-*psbJ*, *psbM*-*trnD-GUC*, *ndhC*, *matK* and *rps3*-*rpl22*) were suggested as DNA barcodes in other plant groups, such as Zingiberaceae species, Coffeeae alliance, Cinnamomeae, and *Clethra fargesii* Franch. and *Peucedanum* L*.* [22, 33–36]. Although these aforementioned markers have relatively large variability, the impact of gene-level heterogeneity on the phylogenetic reconstruction of plastids should be considered [37].

### Phylogenetic relationships within *Lysimachia*

*Lysiamchia* s.l., newly circumscribed to include all satellite genera (i.e., *Anagallis*, *Trientalis*, *Glaux*, *Asterolinon* and *Pelletiera*) [11, 15], contains approximately 250 species [10]. Previous studies have recovered eleven major clades of *Lysimachia* [2, 8, 10, 12], and its backbone phylogeny has been well resolved except for two nodes (i.e., the positions of the *L. maritima* and *Anagallis* clades; see Yan et al. [10]). In this study, eight major clades were recovered, which was concordant with the results found in several previous studies [2, 8, 10–12]. However, three other major clades were not included in this study. The plastome trees showed that most nodes in each clade were largely improved and strongly supported (Fig. 4a) when compared with those based on a few plastid markers (e.g., [10]). The weakly supported relationships were mainly in clade I and clade II (Figs. S7-10), which were likely caused by limited phylogenetic signals and extensive phylogenetic conflicts due to gene-level heterogeneity in *Lysimachia* plastomes (Figs. S11-12), suggesting that the two clades may have experienced a short radiation period from their most recent common ancestor (MRCA).

In this study, the phylogenetic positions of several *Lysimachia* species were of interest. *Lysiamchia coreana*, an endemic taxon in Korea, belongs to clade V. This species was first described by Takenoshin Nakai in 1909 [38]. Morphologically, *L. coreana* is similar to *L. davurica*, as confirmed by palynotaxonomic data [39]. However, recent study has treated *L. coreana* as a synonym of *L. davurica* [40], suggesting that it should be classified under subg. *Lysimachia* (Vulgaris clade, IV clade). Our results, however, revealed that *L. coreana* forms a separate clade (clade V) and does not cluster with *L. davurica* and *L. vulgaris* of subg. *Lysimachia* (Vulgaris clade, clade IV) (Fig. 4). The plastome of *L. coreana* used in this study was obtained from GenBank [25], which made it difficult to determine whether the observed differences were due to the quality of the sequencing data or inherent species differences. If the latter is the case, it is appropriate to consider *L. coreana* as a distinct species.

Furthermore, it is imperative to conduct further investigations on the phylogenetic position of *L. fortunei*. This species, belonging to subgenus *Palladia*, is extensively distributed in central and southeastern China, Vietnam, the Korean peninsula, and Japan [1]. Despite its widespread distribution, there has been little controversy regarding the taxonomic classification of *L. fortunei* since its initial description in 1868 [1]. Morphologically, *L. fortunei* bears the closest resemblance to *Lysimachia chikungensis* Bail., but differs in its creeping rhizomes and relatively large leaves [1]. Unfortunately, we were unable to test the hypothesis of their close relationship due to the absence of *L. chikungensis* accessions in this study. Our findings indicate that two *L. fortunei* accessions did not cluster together (Fig. 4a, Figs. S7-10). This scenario may be attributed to three factors. Firstly, the wide range of habitats and ecological niches occupied by *L. fortunei* may have resulted in significant differentiation at the intraspecific taxonomic level, suggesting the possibility of cryptic lineages within this species. Secondly, misidentification may have occurred, as one accession (MW455171) was obtained directly from GenBank, and we were unable to examine its voucher specimens. Finally, hybridization/introgression is a common occurrence in plants [41], and chloroplast capture is frequently observed during hybridization/introgression events [42]. Therefore, hybridization/introgression may be a plausible explanation for the paraphyly of *L. fortunei* accessions. In conclusion, the taxonomic status of *L. fortunei* warrants further examination through the use of additional samples and genetic markers, such as low-copy nuclear markers, in future studies.

### The phylogenetic position of *Lysimachia maritima* and its presumed origin history

*Lysimachia maritima* (formerly belonging to the monotypic genus *Glaux*; clade III) is undoubtedly a member of *Lysimachia*, as supported by molecular systematics [2, 15, 43]. However, its phylogenetic position remains contentious. Yan et al. [10] recovered *L. maritima* (clade III) as sister to subg. *Palladia* (clade II) + subg. *Lysimachia* (clade I) with weak support, based on ten plastid markers and ITS. When only ITS data was used, *L. maritima* was recovered as sister to either subg. *Palladia* [12] or subg. *Lysimachia* [8]. In the present study, plastid ML trees and species tree strongly supported *L. maritima* (clade III) as a sister to subg. *Palladia* (clade II) + subg. *Lysimachia* (clade I), while the nrDNA ML tree clearly placed *L. maritima* (clade III) and subg. *Palladia* (clade II) as a sister group (Fig. 4a; Figs. S7-10).

Plastid loci have traditionally been concatenated into a single dataset for phylogenetic inference due to the low levels of recombination in organelle genomes; therefore, their different historical signals that lead to incongruence between nucleotide characters or sequence blocks have long been assumed to be negligible ([44], and references therein). However, recent studies [45–49] have challenged this view, although the sources of conflict within the plastome remain understudied and poorly understood [46, 47, 50], such as heterogeneity in molecular evolution, heteroplasmy, and recombination. Therefore, the cytonuclear discordance detected in this study was first examined within the plastome to better characterize the extent and sources of the conflict signature. Quartet sampling and phyparts analyses both support Clade III (*L. maritima*) as a sister to Clades I and II (Figs. S11-12). Furthermore, 28 of 156 plastid loci had phylogenetic signals at the controversial node (clades I-III), of which only five loci significantly supported (△*ln*L > 2) the opposite constrained tree (T5) (Fig. 4b, c; Table S5). This suggests that the original plastid relationship (clade III, (clade I, clade II)) is robust. It is plausible that heterogeneity in molecular evolution may have resulted in a few plastid fragments that have generated opposing topologies.

It is well-documented that inconsistency between plastid and nuclear gene trees is a common and widespread phenomenon in plants [51]. Previous studies have suggested that certain evolutionary events, such as gene replication, hybridization, and lineage sorting of ancestral polymorphisms, may be responsible for the incongruent topologies from the plastome and nuclear genome [42, 51, 52]. The pattern of longer internal edges followed by short external edges of the node of *L. maritima* (clade III) and subg. *Palladia* (clade II) in the nrDNA ML tree (Fig. 4a) is consistent with the pattern of a relatively constant rate of diversification [53], coupled with low ILS level due to reduced gene tree discordance and quartet incongruence (Figs. S11-12), suggesting the possibility of hybridization rather than incomplete lineage sorting (ILS) on the early diversification of the group (clades II-III).

*Lysimachia maritima* undoubtedly occupies an isolated position within *Lysimachia* due to its corolla loss and specialized adaptation to saline habitats. Morphologically, it is similar to *L. mauritiana* of subg. *Palladia* but lacks a corolla [2], suggesting a potential relationship between the two taxa. Our nuclear data strongly indicate that *L. maritima* is closely related to subg. *Palladia*, while the plastome suggests that the species is a more independent lineage. It is hypothesized that the plastome of *L. maritima* is derived from a lineage that is either extinct or has not been sampled (a “ghost lineage”; [54, 55]). Its hybrid origin may have resulted in adaptive introgression, which may have enabled *L. maritima* to acquire its unique morphological characteristics (such as the corolla lost) and habitat adaptation (i.e., typically found on saline soils, such as beaches, muddy shallows, saline soils, and inland salt marshes). However, only one nuclear gene, nrDNA, was used for the construction of the nuclear gene tree in this study, so we cannot rule out the possibility that systematic errors in nrDNA itself caused this plastid-nuclear discordance, which should be tested further with more nuclear data.

## Conclusions

In this study, the plastomes of 50 *Lysimachia* taxa were comparatively analyzed. Generally, the structures of these plastomes were conserved, although contraction and expansion of the IR regions was observed to be associated with plastome sequence variation. Thirteen hotspot regions were identified within *Lysimachia* plastomes, which could potentially serve as plastid markers for the identification of *Lysimachia* species. Additionally, signatures of positive selection were detected in *ndhB*, *petB* and *ycf2* in *Lysimachia*, subg. *Palladia*, and subg. *Lysimachia,* respectively, suggesting that these genes may be involved in adaptation and speciation processes in *Lysimachia*. These genomic data provided new insights into the interspecific relationships of *Lysimachia*, including the identification of a cytonuclear discordance of the position of *L. maritima*, which may be the result of ghost introgression in the past. Our findings have established a basis for further exploration of the taxonomy, phylogeny and evolutionary history within *Lysimachia*.

## Materials and methods

### Sampling, DNA extraction, and sequencing

A total of 50 *Lysimachia* individuals, representing 46 species, were included in the present study. Forty-three of these *Lysimachia* individuals were newly sampled, with fresh and healthy leaves collected in the field and dried with silica gel for DNA extraction. Voucher specimens were formally identified by Prof. Chi-Ming Hu, Gang Hao and Hai-Fei Yan and deposited at the Herbarium of South China Botanical Garden (IBSC). Additionally, seven *Lysimachia* plastomes were obtained from GenBank (i.e., *L. coreana*, *L. congestiflora* Hemsl., *L. christinae* Hance, *L. maritima*, *L. fortunei*, *L. candida* Lindl., and *L. mauritiana*). Although the sampling ratio of the genus was low (*c*. 18%), it represented all main clades in China identified by previous studies, except subg. *Naumburgia* [2, 8, 10]. Furthermore, the majority of our sampling (*c*. 93%) belonged to three highly diversified subgenera of *Lysimachia* s.s. in East Asia [10], namely 24, 14, and 5 species from subgenera *Lysimachia*, *Palladia*, and *Idiophyton*, respectively, each accounting for approximately 32%, 23%, and 8% of the above subgenera. Plastomes of five representatives of Primulaceae (i.e., *Ardisia crenata* Sims., *Myrsine stolonifera* (Koidzumi) E. Walker, *Aegiceras corniculatum* (L.) Blanco, *Embelia vestita* Roxb. and *Primula poissonii* Franch.) were downloaded from GenBank and used as outgroups for plastid phylogenetic analyses. For nrDNA phylogenetic inferences, nrDNA sequences of *Ar. japonica* (Thunb.) Blume, *Ae. corniculatum* and *P. veris* L. were obtained from GenBank as outgroups. The sampling details can be found in Table S1.

### DNA extraction, sequencing, assembly and annotation

Total genomic DNA was extracted from leaf materials using a modified cetyltrimethylammonium bromide (CTAB) method [56]. Genomic DNA was randomly fragmented into 350-bp fragments for constructing pair-end libraries and sequenced on MGISEQ-2000 (MGI, Shenzhen, China) at the Beijing Genomics Institution (BGI, Wuhan, China). Approximately 2 Gb of genome skimming data were generated for each sample. Paired-end sequence reads were trimmed to remove low-quality reads and adapter sequences using Trimmomatic v0.36 [57] before assembly. Plastomes were assembled using the GetOrangelle v1.7.7.0 [58] with default parameters. The completed assembled plastiome sequences were checked and adjusted manually in Geneious v11.0.3 [59] and were annotated by the online annotation program GeSeq v2.03 [60]. A graphical circular plastome map representing *Lysimachia* was drawn using OGDRAW v1.3.1 [61, 62].

To confirm whether phylogenetic conflicts between the plastid and nuclear genomes in *Lysimachia* existed, the complete nrDNAs of 40 taxa were assembled based on the above genome skimming data by GetOrangelle with the parameters set as R = 15 and K = 21, 45, 65, 85, 105 and annotated and checked in Geneious. The whole nrDNA is composed of the intergenic spacer (IGS), the small-subunit (SSU) ribosomal RNA (rRNA) gene, the internal transcribed spacer 1 (ITS1), the 5.8S rRNA gene, the internal transcribed spacer 2 (ITS2), and the large-subunit (LSU) rRNA gene. The annotated plastomes and nrDNA sequences were deposited in GenBank with accession numbers (Table S1).

### Comparative analyses, mutation hotspot identification, and substitution rate estimations

The boundary information between IRs and SSC/LSC was analyzed in Geneious. The relationship between the plastome size and IR length among species was examined using least squares linear regression in R 4.2.2 [63]. The full interspecific sequence divergences of *Lysimachia* plastomes were visualized using the mVISTA program under the Shuffle-LAGAN model [64], with the *L*. *fortunei* plastome (GenBank accession no.: MW455171) as a reference. The *Lysimachia* plastomes were further aligned using MAUVE v1.1.3 [65] to verify gene orders and structure rearrangements. To identify mutation hotspots, nucleotide diversity (*Pi*) for all protein-coding and noncoding regions was calculated with DnaSP v6 [66] using the sliding window method (window length: 500 bp; step size: 200 bp).

The analysis of evolutionary rate was conducted along the phylogenetic tree of *Lysimachia* for each plastid protein-coding gene. The substitution ratio of nonsynonymous (*d*_N_), synonymous (*d*_S_), and ω (*d*_N_/*d*_S_) values of each protein-coding gene was calculated by a Site Model (M0) using EasyCodeML v1.4 [67]. The ω value is an indicator of selective pressure of the protein-coding genes, and ω > 1, ω = 1, and ω < 1 indicate positive, neutral, and negative selection, respectively. The codon alignment used for this analysis was generated by using the MUSCLE (Codons) option [68] in MEGA 11 [69]. The fasta format sequences were converted to “PML” format under the option of the Convert Sequence format in PhyloSuite v1.2.2 [70].

### Phylogenetic analyses and conflicting phylogenetic signal test

Fifty-five plastomes were extracted and aligned for all annotated loci, including coding and noncoding regions. Sites with > 80% gaps were trimmed, and loci with alignment lengths greater than 100 bp were retained. Eighty protein-coding genes (PCGs), 92 intergenic spacers (IGSs) and 18 introns were extracted from all plastomes for phylogenetic inferences. Alignments of individual coding or noncoding regions were generated using MAFFT v.7.4 [71] with a default setting. The concatenated alignments for four multigene datasets were generated by using the AMAS package [72]. RAxML v.8 [73] was used for plastid phylogenetic analyses under the GTRGAMMA model with the “autoMRE” option, which generates bootstrap replicates until the support values converge.

The multiple species coalescent model was used to infer phylogenetic relationships, which accounts for genealogic heterogeneity and allows the assessment of ancient hybridization/introgression and ILS [74]. Gene trees of the 190 plastid loci inferred by RAxML with the GTRGAMMA model and autoMRE rapid bootstraps. Poorly supported branches (i.e., bootstrap support (BS) < 50%) in each gene tree were collapsed. Maximum Quartet Support Species Tree (MQSST) analyses were performed under the coalescent model with plastid gene trees in ASTRAL‐III v.5.6.3 [75]. Internal branch supports of the species tree were evaluated with local posterior probabilities (LPPs; [76]) by ASTRAL and with the quartet sampling (QS) method based on 1000 replicates per branch [77]. The quartet sampling developed four metrics to evaluate the quartet concordance (QC), differential (QD), and informativeness (QI) for each branch and fidelity (QF) scores for each terminal in the given phylogeny. Phyparts [78] was used to assess whether gene tree topologies were concordant or conflicting with the species tree. For each node on the species tree, the ratio of concordant and conflicting or no support loci was visualized by a pie chart.

We conducted topology selection analyses with five maximum likelihood (ML) trees using the whole plastid dataset. The weighted Kishino-Hasegawa test (wKH test), weighted Shimodaira-Hasegawa test (wSH test) and Approximately Unbiased test (AU test) were used to examine the significant difference between the five ML tree topologies (the whole plastid tree (T1), PCG tree (T2), IGS tree (T3), intron tree (T4) and constrained plastid tree conforming to the hypothesis of Clade III in nrDNA phylogeny (T5)) in IQ-TREE [79]. The best-fit model of the five datasets according to Bayesian Information Criterion (BIC) was chosen as TVM + F + I + I + R5. Furthermore, the cytonuclear discordances between the plastome and nrDNA were examined. To detect the conflicting signals of two given hypotheses inside the plastome, we compared the log-likelihood scores between the constrained and unconstrained treatments following the method of Shen et al. [80]. We obtained the constrained tree using the option “-g” in RAxML and computed per locus log-likelihood values for the two trees with the option “-f g” in RAxML. The *ln*L differences between two hypotheses for each locus were ranked and plotted by the ggplot2 package [81] in R. An absolute value of *ln*L difference greater than 2 (△*ln*L > 2) was considered a strong signal for the support of the main hypothesis. We also removed the top five loci in the △*ln*L ranking list and reconstructed the ML tree with the reduced plastid dataset to test the robustness of the plastid phylogeny.

## Supplementary Information

**Additional file 1:** **Fig. S1.** Comparison of LSC, IRs, and SSC junction positions among *Lysimachia* plastomes.

**Additional file 2: Fig. S2.** Correlation of intron and IGS length and *Lysimachia* plastome size. (a) Correlation of intron length and *Lysimachia* plastome size. (b) Correlation of IGS length and *Lysimachia* plastome size.

**Additional file 3: Fig. S3.** Mauve alignment of 50 representative *Lysimachia* plastomes.

**Additional file 4:** **Fig. S4.** Sequence identity plots of plastomes of *Lysimachia* by mVISTA using *Lysimachia fortunei* as the reference. The top line shows the orientation of genes. A cutoff of 70% identity was used for the plots, and the Y-scale represents the percentage identity ranging from 50 to 100%.

**Additional file 5:** **Fig. S5.** Comparison of nucleotide diversity (*Pi*) values in plastomes of four main *Lysimachia* clades. (a) The subg. *Palladia *(clade III). (b) The subg. *Idiophyton *(clade VI). (c) The subg. *Lysimachia* (clade I). (d) The subg. *Lysimachia* (clade IV).

**Additional file 6:** **Fig. S6**. Comparisons of *d*_N_, *d*_S_, and *d*_N_/*d*_S_ of the protein-coding genes in three main subg. *Lysimachia* clades. (a) The 53 protein-coding genes in subg. *Palladia*. (b) The 49 protein-coding genes in subg. *Idiophyton*. (c) The 51 protein-coding genes in subg. *Lysimachia* (Christinae clade).

**Additional file 7:** **Fig. S7.** The maximum likelihood tree (T2) of *Lysimachia* reconstructed based on PCGs. The bootstrap support values are indicated along the branches.

**Additional file 8:** **Fig. S8.** The maximum likelihood tree (T3) of *Lysimachia* reconstructed based on IGS. The bootstrap support values are indicated along the branches.

**Additional file 9:** **Fig. S9.** The maximum likelihood tree (T4) of *Lysimachia* reconstructed based on introns. The bootstrap support values are indicated along the branches.

**Additional file 10:** **Fig. S10.** The maximum quartet support species tree (MQSST) inferred by ASTRAL based on plastid regions. Local posterior probabilities (LPPs) are indicated along the branches.

**Additional file 11:** **Fig. S11.** The results of quartet sampling analysis are based on the ASTRAL species tree using the whole plastid dataset. The three metrics are shown on each branch following the order of QC/QD/QI scores (QC: quartet concordance; QD: quartet differentiation; QI: quartet information). The QI score is a nonlinear elevation metric for quartet supports/conflicts. Heatmap of branches by Quartet Concordance (QC) scores for internal branches: dark green (QC > 0.2), light green (0.2 ≥ QC > 0), and dark orange (QC < −0.05).

**Additional file 12:** **Fig. S12.** Pie charts of the ratio of gene support, conflict, and non-informatic gene loci on each node of an ASTRAL maximum quartet support species tree (MQSST). The blue sector of the pie chart indicates the gene support for the shown topology. The green sector of the pie chart indicates the most common conflicting topologies. The red sector of the pie chart indicates all other supported conflicting topologies. The gray sector has no support for two conflict testing topologies. The number in parentheses represents the most common conflict (green) rather than all other gene tree conflicts (red).

**Additional file 13: Table S1.** Samples, vouchers, and GenBank accessions used in this study. **Table S2.** The plastome features of *Lysimachia* taxa. **Table S3.** Genes annotated of *Lysimachia* plastomes. **Table S4.** Topology tests for ML trees generated by various plastid datasets and constraint tree with the clade III sister to clade II using whole plastid dataset on RAxML. **Table S5.** The difference in log-likelihoods of 156 plastid loci between the two hypotheses T1 and T5.

## Data Availability

All sequences used in this study are available from the National Center for Biotechnology Information (NCBI) (see Table S1).

## Abbreviations

bp: Base pair
CDS: Coding sequence
DNA: Deoxyribonucleic acid
Gb: Gigabases
GC: Guanine-cytosine
kb: Kilobase
ML: Maximum likelihood
MRCA: The most recent common ancestor
RNA: Ribonucleic acid
