# Supplementary figures and images for: Plastome evolution and phylogenomic insights into the evolution of Lysimachia (Primulaceae: Myrsinoideae)
Source: BMC Plant Biol. 2023 Jul 14;23:359. doi: 10.1186/s12870-023-04363-z (PMC10347800; doi:10.1186/s12870-023-04363-z)

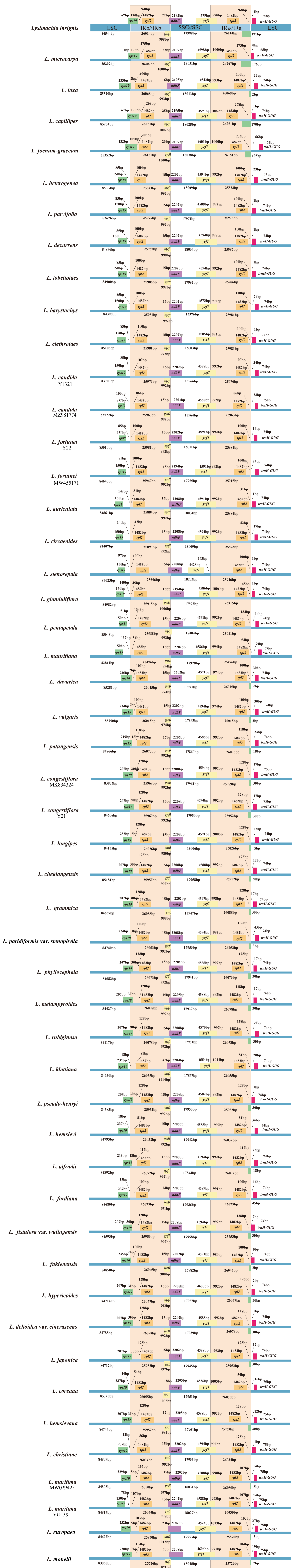

Supplement: Supplementary file 1 — Additional file 1: Fig. S1. Comparison of LSC, IRs, and SSC junction positions among Lysimachia plastomes. [file 12870_2023_4363_MOESM1_ESM.pdf]

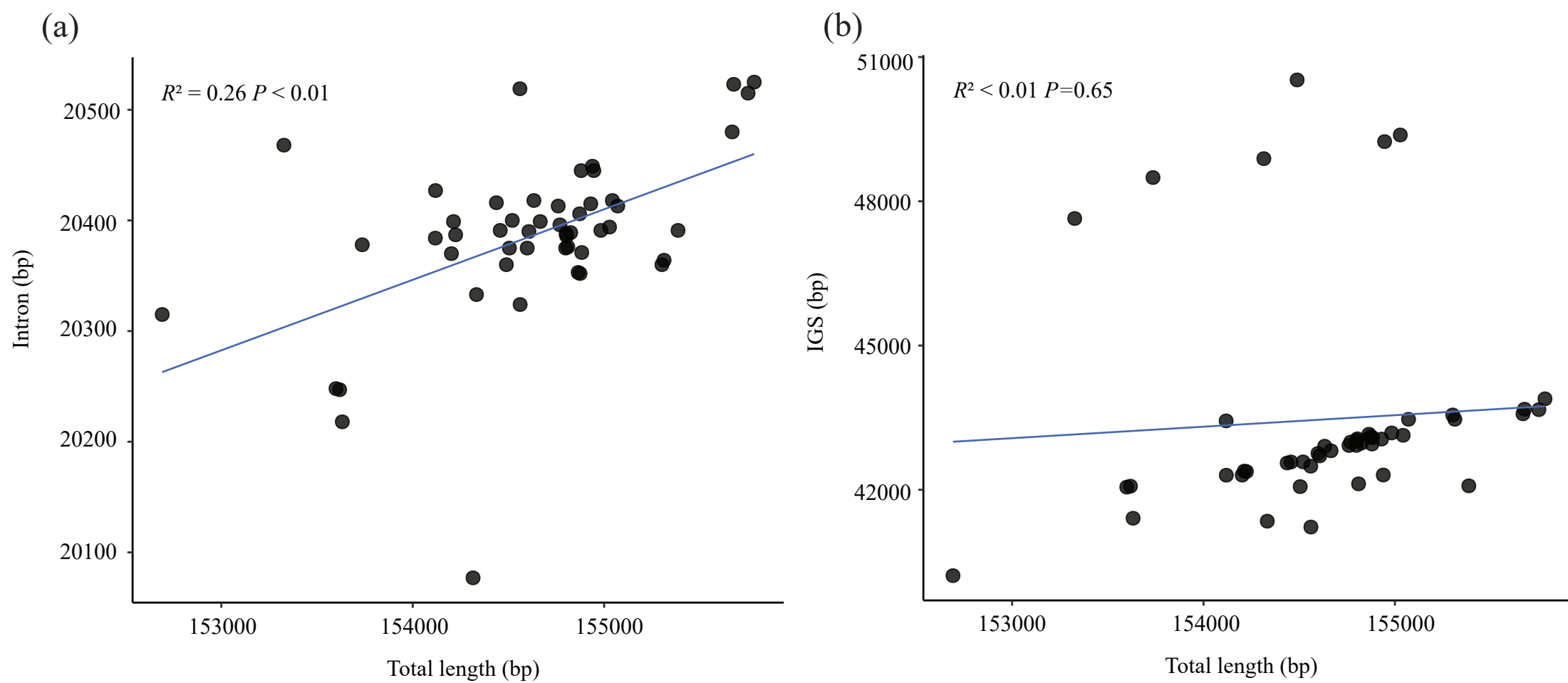

Fig. S2 Correlation of intron and IGS length and *Lysimachia* plastome size.

Supplement: Supplementary file 2 — Additional file 2: Fig. S2. Correlation of intron and IGS length and Lysimachia plastome size. (a) Correlation of intron length and Lysimachia plastome size. (b) Correlation of IGS length and Lysimachia plastome size. [file 12870_2023_4363_MOESM2_ESM.pdf]

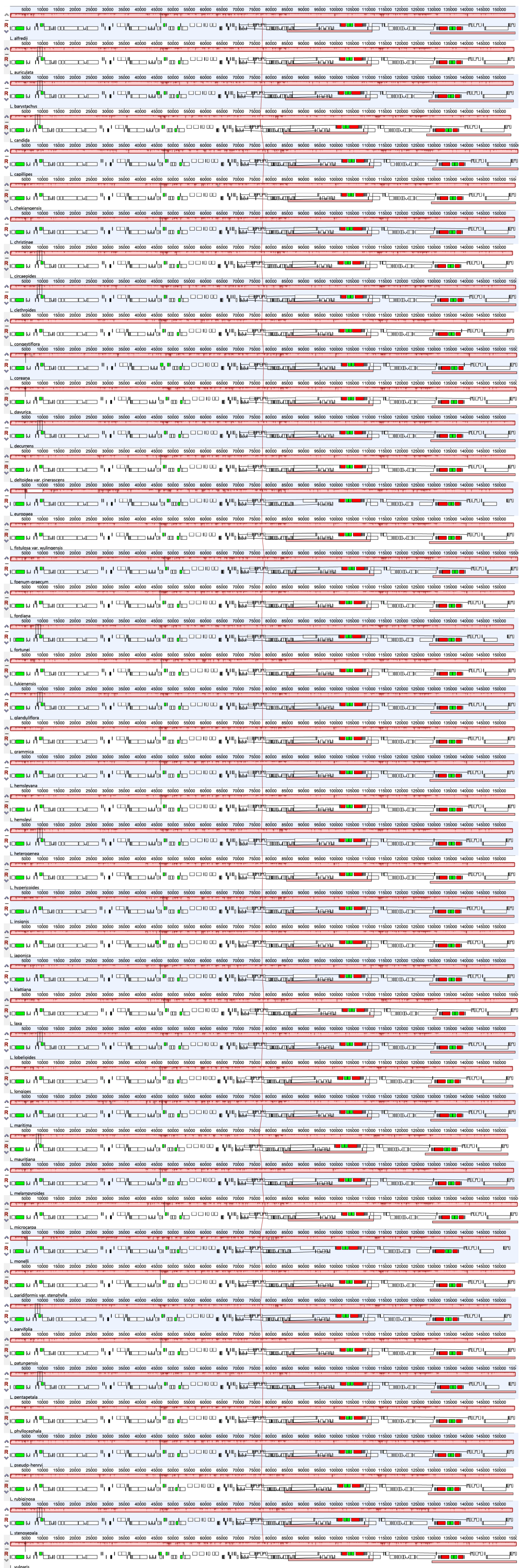

Fig. S3 Mauve alignment of 50 representative *Lysimachia* plastomes.

Supplement: Supplementary file 3 — Additional file 3: Fig. S3. Mauve alignment of 50 representative Lysimachia plastomes. [file 12870_2023_4363_MOESM3_ESM.pdf]

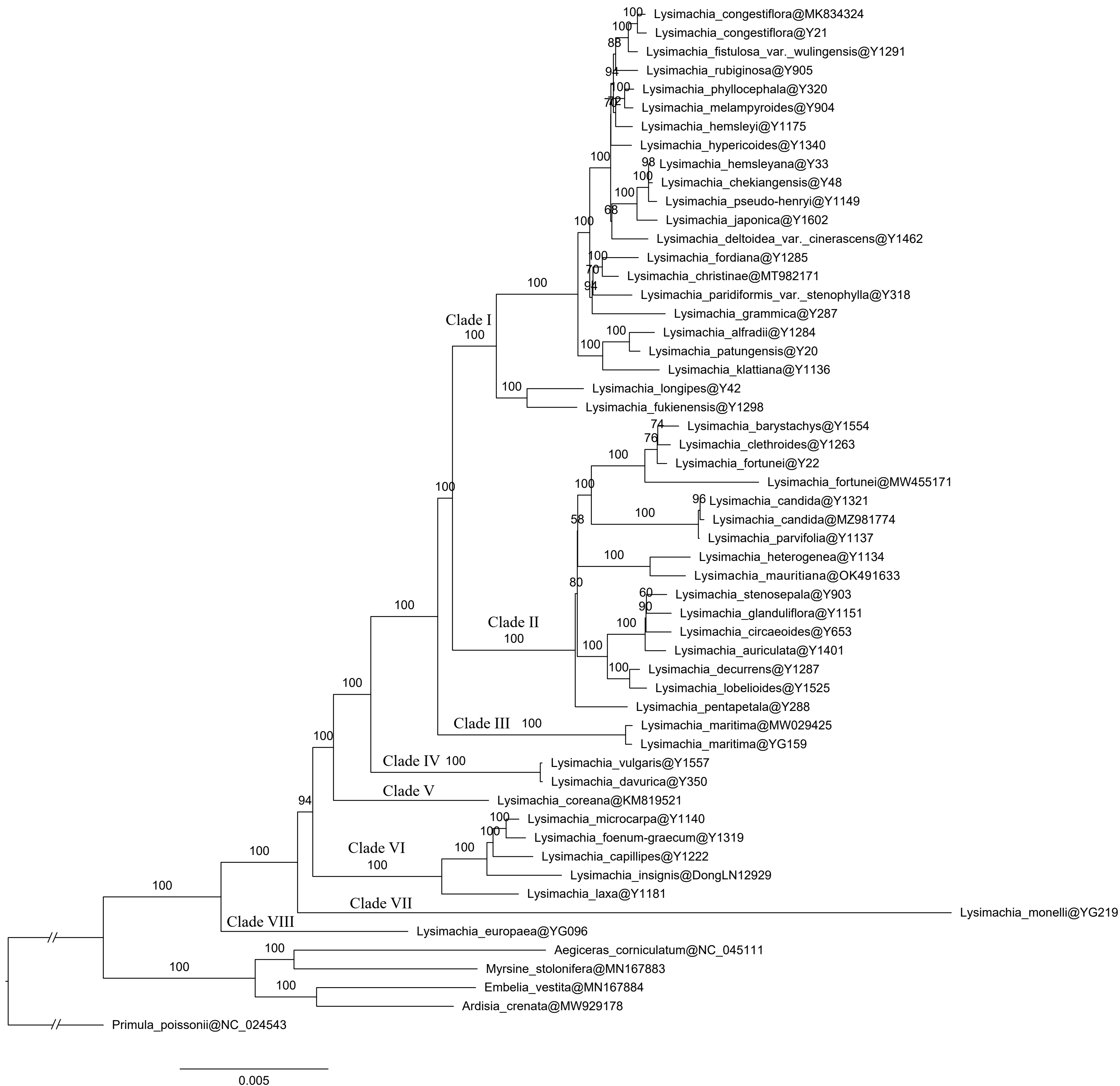

Fig. S7 The maximum likelihood tree (T2) of *Lysimachia* reconstructed based on PCGs.

Supplement: Supplementary file 7 — Additional file 7: Fig. S7. The maximum likelihood tree (T2) of Lysimachia reconstructed based on PCGs. The bootstrap support values are indicated along the branches. [file 12870_2023_4363_MOESM7_ESM.pdf]

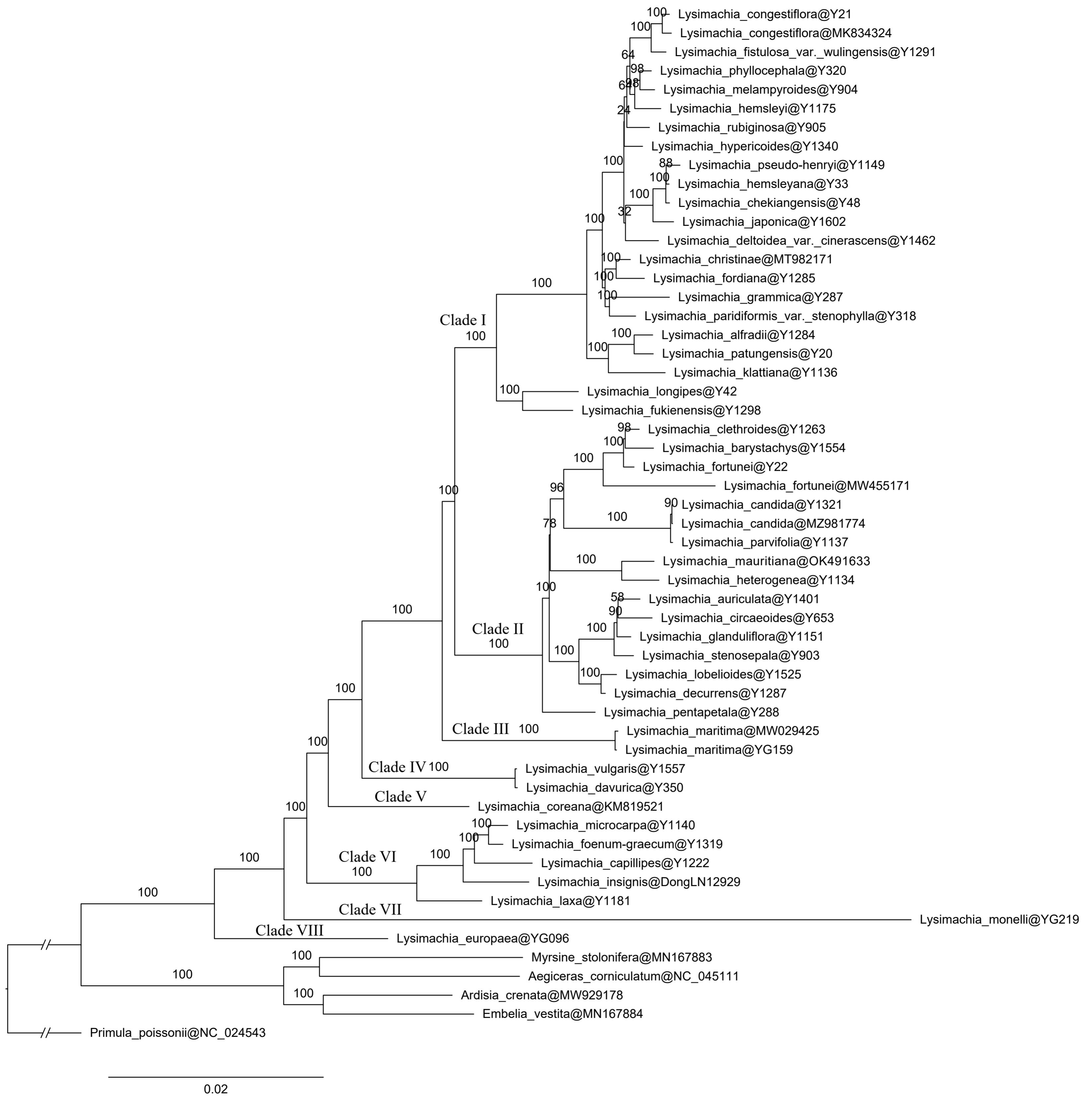

Fig. S8 The maximum likelihood tree (T3) of *Lysimachia* reconstructed based on IGS.

Supplement: Supplementary file 8 — Additional file 8: Fig. S8. The maximum likelihood tree (T3) of Lysimachia reconstructed based on IGS. The bootstrap support values are indicated along the branches. [file 12870_2023_4363_MOESM8_ESM.pdf]

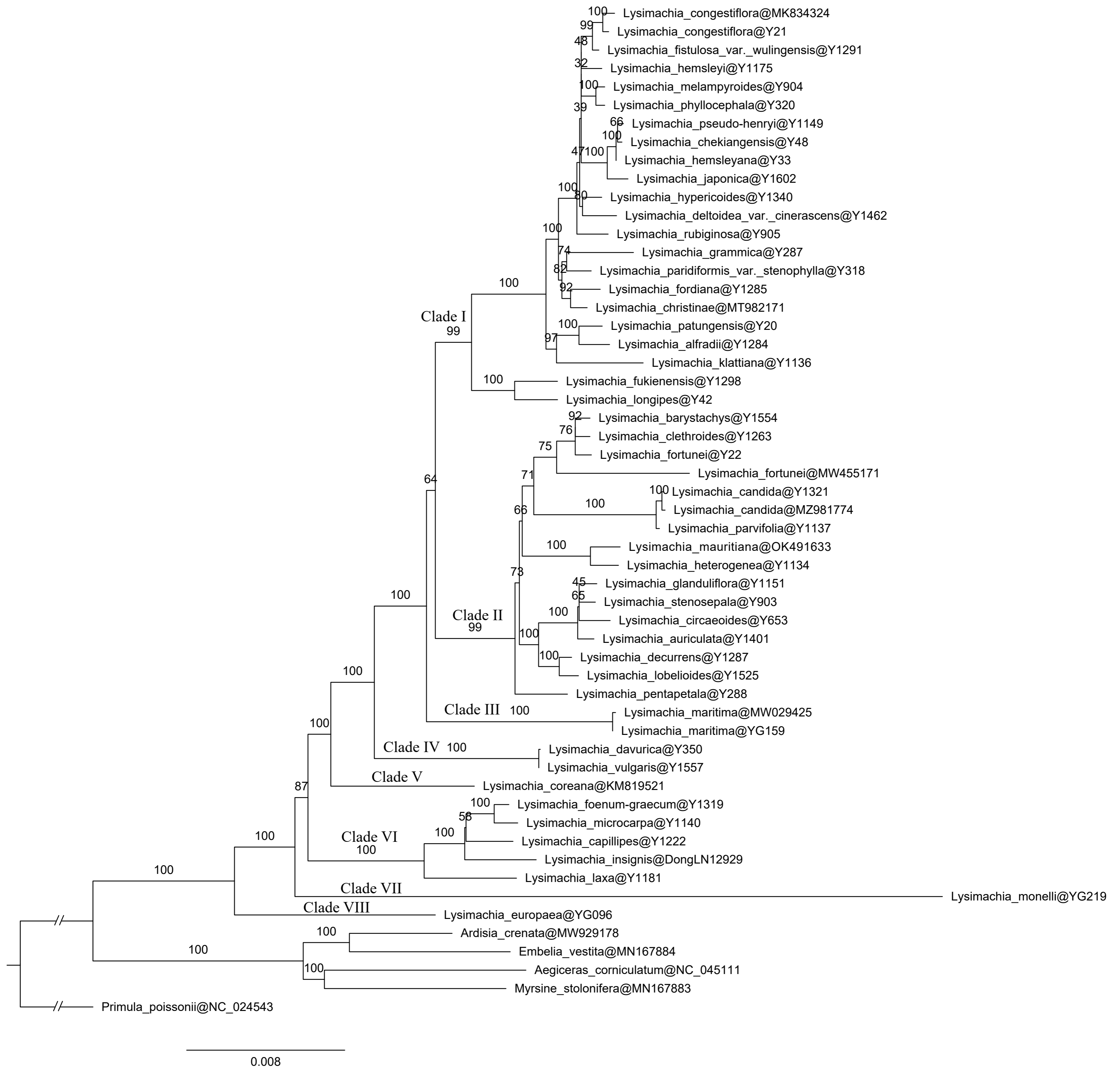

Fig. S9 The maximum likelihood tree (T4) of *Lysimachia* reconstructed based on introns.

Supplement: Supplementary file 9 — Additional file 9: Fig. S9. The maximum likelihood tree (T4) of Lysimachia reconstructed based on introns. The bootstrap support values are indicated along the branches. [file 12870_2023_4363_MOESM9_ESM.pdf]
