# Supplementary material for: Plastome evolution and phylogenomic insights into the evolution of Lysimachia (Primulaceae: Myrsinoideae)
Source: BMC Plant Biol. 2023 Jul 14;23:359. doi: 10.1186/s12870-023-04363-z (PMC10347800; doi:10.1186/s12870-023-04363-z)
Supplement: Supplementary file 4 — Additional file 4: Fig. S4. Sequence identity plots of plastomes of Lysimachia by mVISTA using Lysimachia fortunei as the reference. The top line shows the orientation of genes. A cutoff of 70% identity was used for the plots, and the Y-scale represents the percentage identity ranging from 50 to 100%. [file 12870_2023_4363_MOESM4_ESM.pdf]

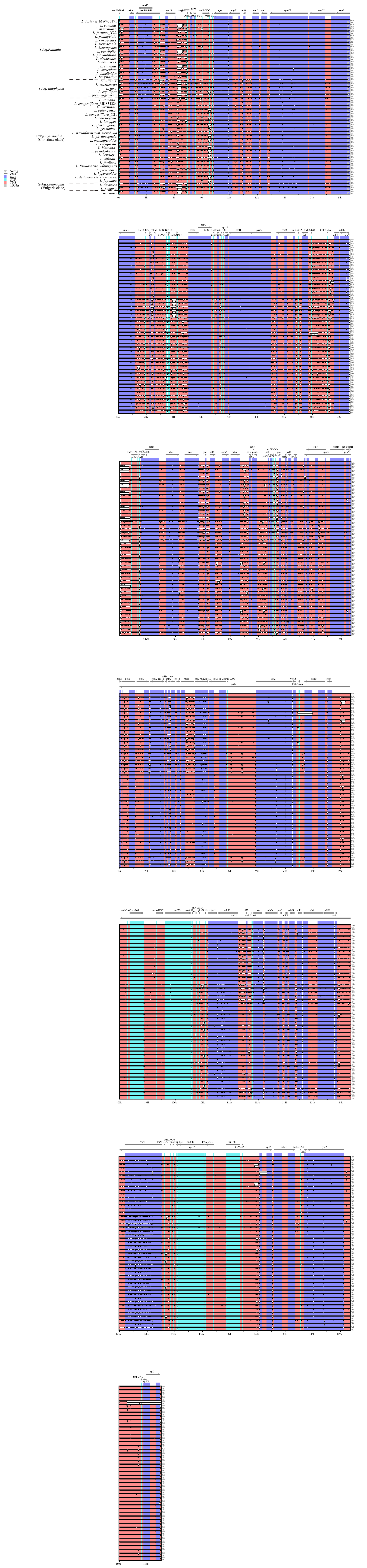

Fig. S4 Sequence identity plots of plastomes of *Lysimachia* by mVISTA using *Lysimachia fortunei* as the reference.
