# Supplementary material for: Plastome evolution and phylogenomic insights into the evolution of Lysimachia (Primulaceae: Myrsinoideae)
Source: BMC Plant Biol. 2023 Jul 14;23:359. doi: 10.1186/s12870-023-04363-z (PMC10347800; doi:10.1186/s12870-023-04363-z)
Supplement: Supplementary file 5 — Additional file 5: Fig. S5. Comparison of nucleotide diversity (Pi) values in plastomes of four main Lysimachia clades. (a) The subg. Palladia (clade III). (b) The subg. Idiophyton (clade VI). (c) The subg. Lysimachia (clade I). (d) The subg. Lysimachia (clade IV). [file 12870_2023_4363_MOESM5_ESM.pdf]

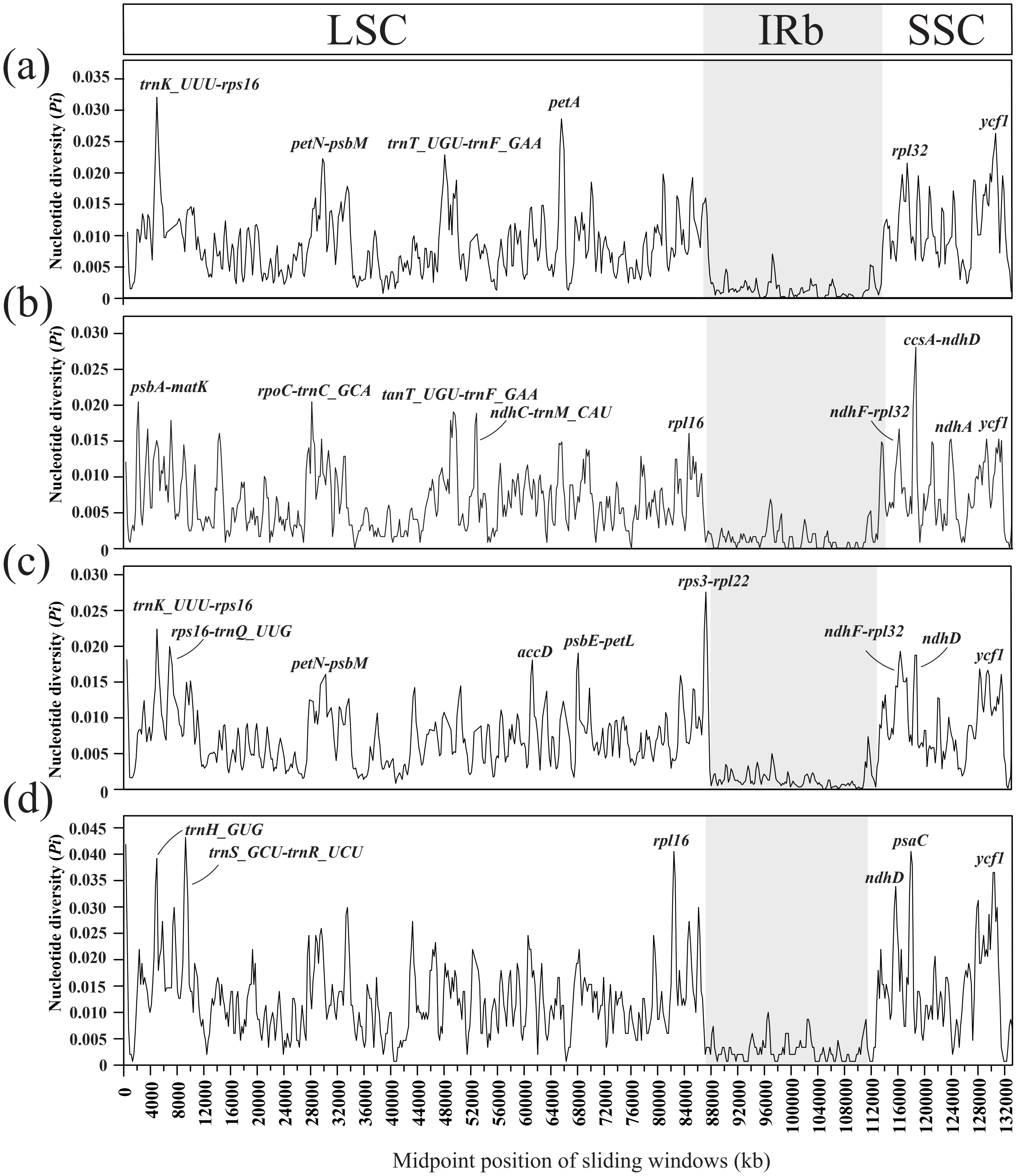

Fig. S5 Comparison of nucleotide diversity (*Pi*) values in plastomes of four main *Lysimachia* clades.
