# Supplementary material for: Plastome evolution and phylogenomic insights into the evolution of Lysimachia (Primulaceae: Myrsinoideae)
Source: BMC Plant Biol. 2023 Jul 14;23:359. doi: 10.1186/s12870-023-04363-z (PMC10347800; doi:10.1186/s12870-023-04363-z)
Supplement: Supplementary file 6 — Additional file 6: Fig. S6. Comparisons of dN, dS, and dN/dS of the protein-coding genes in three main subg. Lysimachia clades. (a) The 53 protein-coding genes in subg. Palladia. (b) The 49 protein-coding genes in subg. Idiophyton. (c) The 51 protein-coding genes in subg. Lysimachia (Christinae clade). [file 12870_2023_4363_MOESM6_ESM.pdf]

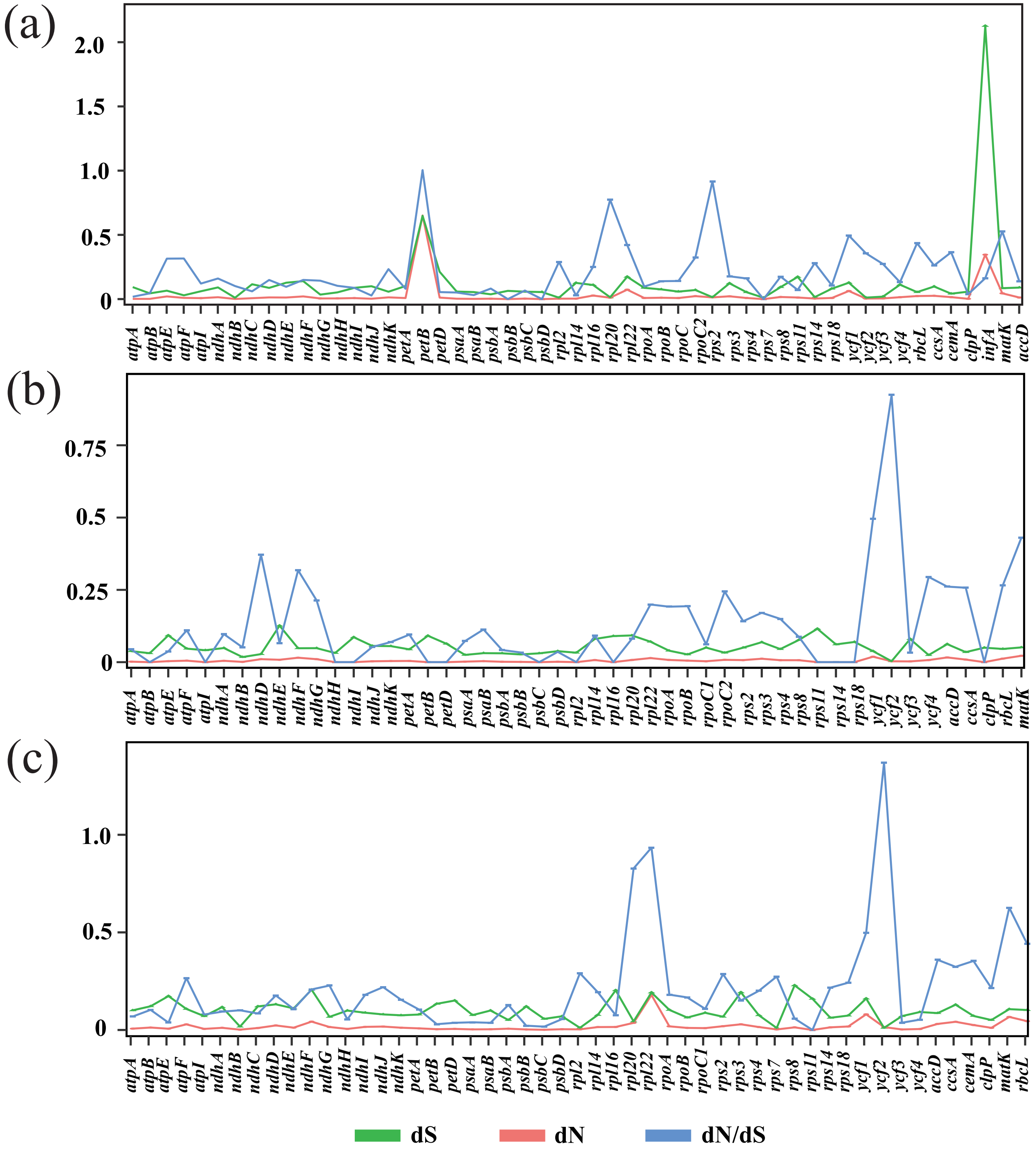

Fig. S6 Comparisons of dN, dS, and dN/dS of the protein-coding genes in three main subg. *Lysimachia* clades.
