# Supplementary material for: Plastome evolution and phylogenomic insights into the evolution of Lysimachia (Primulaceae: Myrsinoideae)
Source: BMC Plant Biol. 2023 Jul 14;23:359. doi: 10.1186/s12870-023-04363-z (PMC10347800; doi:10.1186/s12870-023-04363-z)
Supplement: Supplementary file 10 — Additional file 10: Fig. S10. The maximum quartet support species tree (MQSST) inferred by ASTRAL based on plastid regions. Local posterior probabilities (LPPs) are indicated along the branches. [file 12870_2023_4363_MOESM10_ESM.pdf]

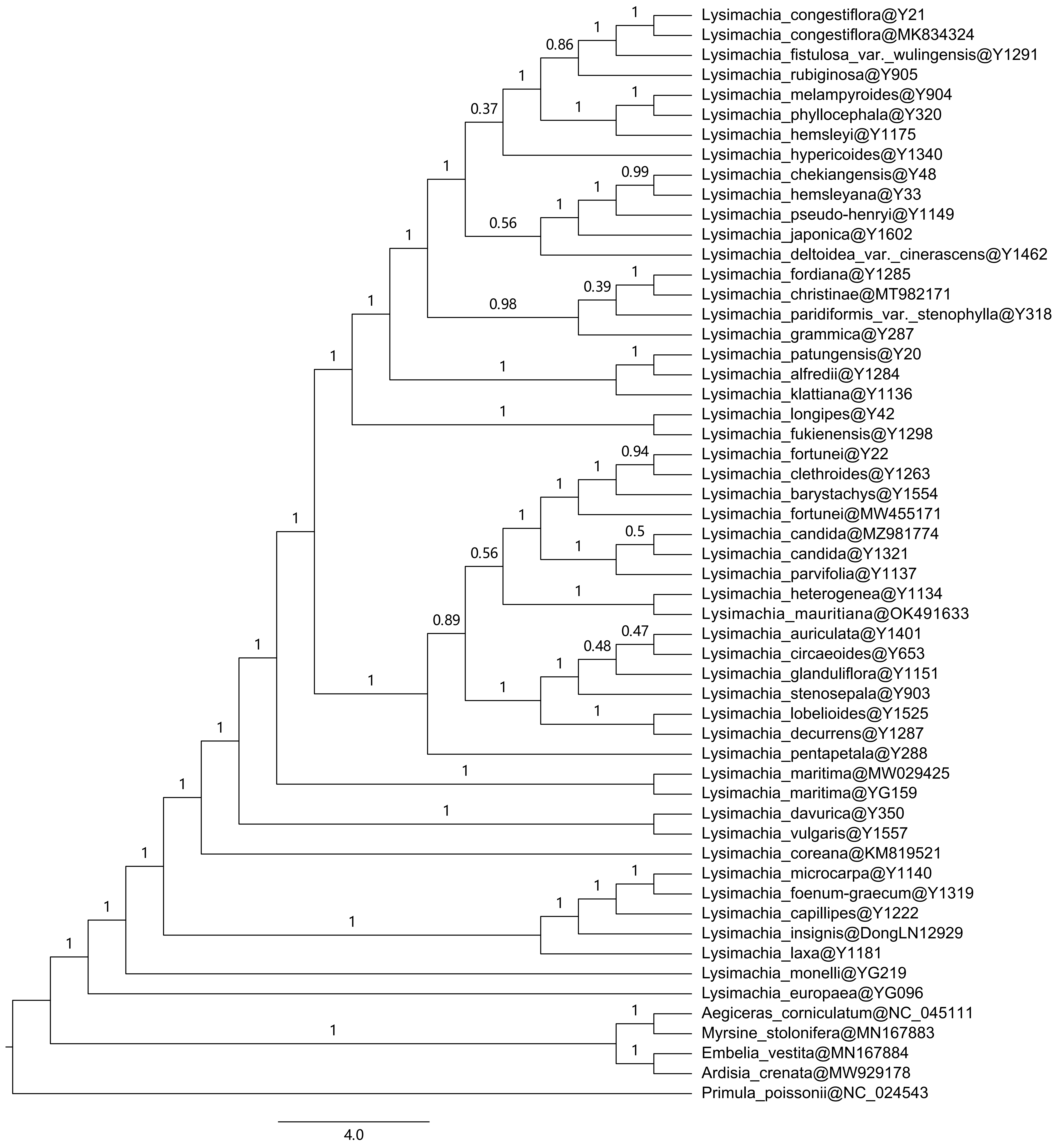

Fig. S10 The maximum quartet support species tree (MQSST) inferred by ASTRAL based on plastid regions.
