# Supplementary material for: Plastome evolution and phylogenomic insights into the evolution of Lysimachia (Primulaceae: Myrsinoideae)
Source: BMC Plant Biol. 2023 Jul 14;23:359. doi: 10.1186/s12870-023-04363-z (PMC10347800; doi:10.1186/s12870-023-04363-z)
Supplement: Supplementary file 11 — Additional file 11: Fig. S11. The results of quartet sampling analysis are based on the ASTRAL species tree using the whole plastid dataset. The three metrics are shown on each branch following the order of QC/QD/QI scores (QC: quartet concordance; QD: quartet differentiation; QI: quartet information). The QI score is a nonlinear elevation metric for quartet supports/conflicts. Heatmap of branches by Quartet Concordance (QC) scores for internal branches: dark green (QC > 0.2), light green (0.2 ≥ QC > 0), and dark orange (QC < −0.05). [file 12870_2023_4363_MOESM11_ESM.pdf]

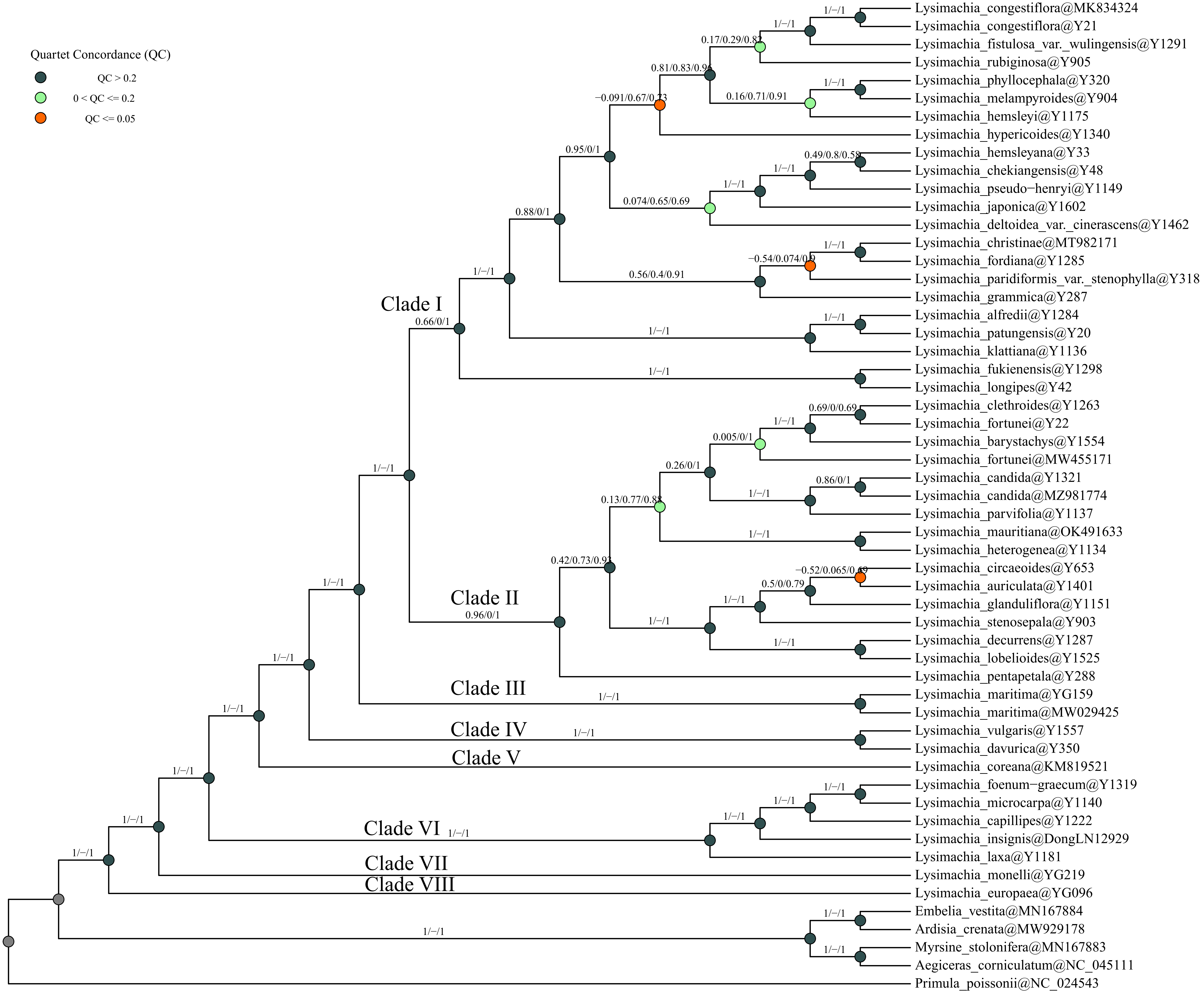

Fig. S11 The results of quartet sampling analysis are based on the ASTRAL species tree using the whole plastid dataset.
