# Supplementary material for: Plastome evolution and phylogenomic insights into the evolution of Lysimachia (Primulaceae: Myrsinoideae)
Source: BMC Plant Biol. 2023 Jul 14;23:359. doi: 10.1186/s12870-023-04363-z (PMC10347800; doi:10.1186/s12870-023-04363-z)
Supplement: Supplementary file 12 — Additional file 12: Fig. S12. Pie charts of the ratio of gene support, conflict, and non-informatic gene loci on each node of an ASTRAL maximum quartet support species tree (MQSST). The blue sector of the pie chart indicates the gene support for the shown topology. The green sector of the pie chart indicates the most common conflicting topologies. The red sector of the pie chart indicates all other supported conflicting topologies. The gray sector has no support for two conflict testing topologies. The number in parentheses represents the most common conflict (green) rather than all other gene tree conflicts (red). [file 12870_2023_4363_MOESM12_ESM.pdf]

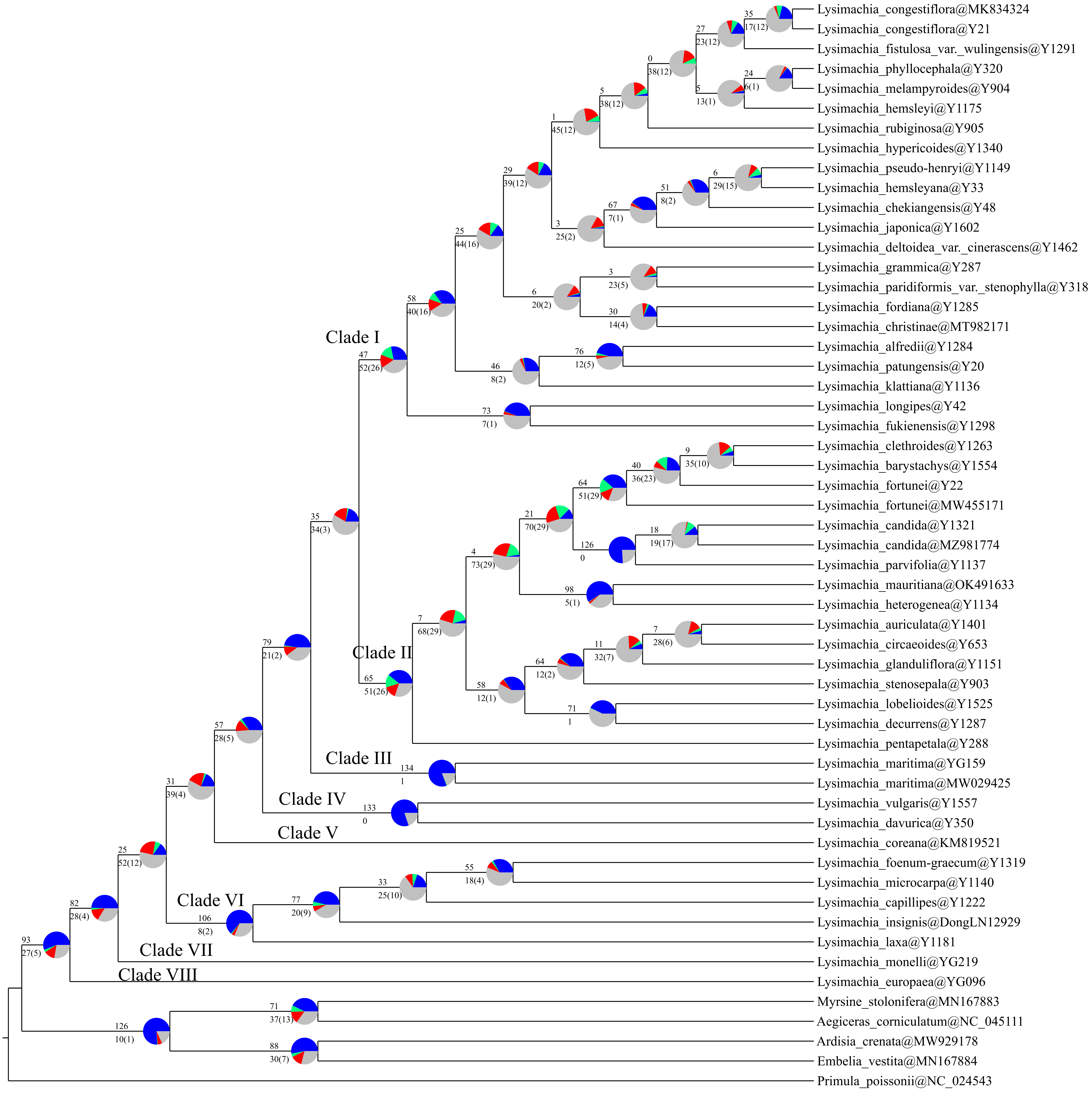

Fig. S12 Pie charts of the ratio of gene support, conflict, and non-informatic gene loci on each node of an ASTRAL maximum quartet support species tree (MQSST).
